# SmMYB113 Is a Key Transcription Factor Responsible for Compositional Variation of Anthocyanin and Color Diversity Among Eggplant Peels

**DOI:** 10.3389/fpls.2022.843996

**Published:** 2022-03-07

**Authors:** Guobin Yang, Lujun Li, Min Wei, Jing Li, Fengjuan Yang

**Affiliations:** ^1^State Key Laboratory of Crop Biology, College of Horticulture Science and Engineering, Shandong Agricultural University, Shandong, China; ^2^Scientific Observing and Experimental Station of Facility Agricultural Engineering (Huang-Huai-Hai Region), Ministry of Agriculture and Rural Affairs, Shandong, China; ^3^Shandong Collaborative Innovation Center for Fruit and Vegetable Production With High Quality and Efficiency, Tai’an, China; ^4^Key Laboratory of Biology and Genetic Improvement of Horticultural Crops (Huanghuai Region), Ministry of Agriculture and Rural Affairs, Shandong, China

**Keywords:** eggplant, peels color, metabolome, anthocyanin biosynthesis, MYB transcription factor

## Abstract

To understand the color formation mechanism in eggplant (*Solanum melongena* L.) peel, a metabolomic analysis was performed in six cultivars with different peel colors. A total of 167 flavonoids, including 16 anthocyanins, were identified based on a UPLC-MS/MS approach. Further analysis revealed that the delphinidins/flavonoids ratio was consistent with the purple coloration of eggplant peels, and *SmF3′5′H* expression level was consistent with the delphinidin 3-O-glucoside and delphinidin 3-O-rutinoside contents, the main anthocyanins in the purple-peels eggplant cultivars identified in this study. *SmMYB113* overexpression promoted anthocyanins accumulation in eggplant peels and pulps. Metabolomic analysis revealed that delphinidins were still the main anthocyanins class in the peels and pulps of *SmMYB113*-OE4, but most anthocyanins were glycosylated at the 5-position of the B-ring. Our results provide new insights into the anthocyanin composition of eggplant peels and demonstrate the importance of *SmMYB113* in stimulating anthocyanin biosynthesis in eggplant fruits.

## Introduction

Anthocyanins are colored water-soluble pigments that give flowers, fruits, and tubers blue, red, or purple coloration. Studies have reported that anthocyanins can have anti-diabetic, anti-cancer, anti-inflammatory, anti-microbial, and anti-obesity effects and prevent cardiovascular diseases (Khoo et al., 2017). Therefore, anthocyanin-rich flowers, fruits, and tubers, such as red rose, blue chicory, purple mint, red cabbage, purple eggplant, and purple potato are popular with consumers.

Recently, eggplant (*Solanum melongena* L.) has received much attention as a functional food. The eggplant extracts have potent antioxidative properties, suggesting their possible involvement in reducing the risk of various disorders (Noda et al., 2000; Sadilova et al., 2006; Akhbari et al., 2019; Condurache et al., 2021). The anthocyanin composition of purple eggplant peels has been reported, however, eggplant peels can be a variety of colors, including black-purple, reddish-purple, lavender, white, orange, or green (Sadilova et al., 2006; Todaro et al., 2009; Ferarsa et al., 2018; Yong et al., 2019; Condurache et al., 2021).

Plants anthocyanins have different grades of glycosylation, hydroxylation, methoxylation, and acylation. Anthocyanidins are the basic anthocyanin structure, and can be divided into six most common types, cyanidin, delphinidin, pelargonidin, peonidin, petunidin, and malvidin. Cyanidin gives plants a reddish-purple (magenta) pigment, while delphinidin appears blue-red or purple. Pelargonidin gives an orange hue to flowers and red to some fruits and berries. Glycosylated cyanidin, delphinidin, and pelargonidin are the most common pigments in nature (Castañeda-Ovando et al., 2009). Peonidin, petunidin, and malvidin are methylated anthocyanidins with the visible color magenta. Peonidin is found abundantly in berries, grapes, and red wines. Malvidin is abundant in blue flowers and red wines. Petunidin has been detected in black currants and purple flowers. The distribution of the six common anthocyanidins in fruits and vegetables is: cyanidin 50%, delphinidin 12%, pelargonidin 12%, peonidin 12%, petunidin 7%, and malvidin 7% (Castañeda-Ovando et al., 2009; Khoo et al., 2017). However, the anthocyanidins antioxidant activities are different. Seeram and Nair (2002) ranked the ability of the six common anthocyanidins to inhibit the Fe(II)-induced lipid peroxidation as delphinidin (70%) > cyanidin (60%) > peonidin (45%) > malvidin (43%) > pelargonidin (40%). Xie et al. (2021) found that delphinidin-3-O-sambubioside has the most potent xanthine oxidase inhibitory activity among 18 anthocyanins examined, and could be used for the prevention and treatment of hyperuricemia. Understanding the anthocyanidin composition of plants can lead to the development of anthocyanin applications to human health.

The technology behind transgenic crops in breeding programs constantly develops and elevates many excellent genotypes. MYB transcription factors (TFs) have been identified to be one of the most important transcriptional regulators in anthocyanins biosynthesis, acting as activators or repressors (Ma and Constabel, 2019; Yan et al., 2021). Anthocyanins content increases when activators MYBs are overexpressed through transient transactivation experiments or transgenic calluses in tobacco leaves and strawberry. Previous studies have reported that *SmMYB113*, orthologous to *AtMYB75*, promote anthocyanins biosynthesis in tobacco leaves in transient expression assays (Li et al., 2017) and eggplant calluses through experimental transformation (Shi S. et al., 2021). However, whether the anthocyanins profile induced by *SmMYB113* is consistent with the original profile in wild-type plants has been rarely reported.

In this study, we report the metabolic profiles of flavonoids that may contribute to different pigmentation in eggplants. Six eggplant cultivars with different peel colors representing all possible eggplant colors were examined (Duan et al., 2021). In addition, the anthocyanin composition of eggplant peels and pulps activated by transgenic overexpression of *SmMYB113* were also investigated using metabolome technology. This study aimed to improve our understanding of the mechanisms of eggplant coloration and to explore whether overexpression of *SmMYB113* alters their anthocyanin profiles.

## Materials and Methods

### Sampling

Six eggplant cultivars (Numbers 44, 64, 76, 108, 109, and 133) (Duan et al., 2021) including five *S. melongena* and one *Solanum aethiopicum* (mainly cultivated and popular in Africa), and *SmMYB113-OE* transgenic eggplants were planted in the solar greenhouse at Shandong Agricultural University. Peels of the six eggplant cultivars and peels and pulps of *SmMYB113-OE* transgenic eggplants were harvested from nine representative fruits of each type in triplicate. The samples were immediately frozen in liquid nitrogen after harvest, and the frozen samples were subjected to anthocyanin content measurement, gene expression analysis, and flavonoids metabolomic analysis.

### Anthocyanins Content Analysis

The anthocyanin contents were extracted using methods detailed in Neff and Chory (1998).

### Ultra Performance Liquid Chromatography-MS/MS Analysis of Flavonoids Metabolomes

Peels or pulps samples were prepared for flavonoid metabolomic analysis according to Chen et al. (2013). The sample extracts were analyzed using the UPLC-MS/MS system (UPLC, Ultra Performance Liquid Chromatography, Shim-pack UFLC SHIMADZU CBM30A system,^1^; MS, Tandem mass spectrometry, Applied Biosystems 6500 QTRAP^2^). The column contained water ACQUITY UPLC HSS T3 C18 (pore size 1.8 μm, length 2.1 × 100 mm). The solvent system contained water (0.04% acetic acid) and acetonitrile (0.04% acetic acid). The gradient program was 95:5 V/V at 0 min, 5:95 V/V at 11.0 min, 5:95 V/V at 12.0 min, 95:5 V/V at 12.1 min, and 95:5 V/V at 15 min. The flow rate was 0.4 ml min^–1^ at 40°C and the injection volume was 2 μl. Data were acquired using multiple reaction monitoring (MRM) with a triple quadrupole tandem mass spectrometer and processed using Analyst 1.6.1 software.

### RNA Extraction and Quantitative Real-Time PCR Analysis

The total RNA was extracted from each sample using TaKaRa MiniBEST Plant RNA Extraction Kit (TaKaRa, Otsu, Shiga, Japan). Next, 1 μg RNA was reverse-transcribed into cDNA using a PrimeScript RT Reagent Kit with gDNA Eraser (TaKaRa). Quantitative real-time PCR (QRT-PCR) analysis was performed using SYBR Premix Ex Taq II Kit (TaKaRa) and LightCycler 96 system (Roche, Basel, Switzerland). The *Actin* gene (GU984779.1) was used as a standard. The relative expression was calculated using the 2^–ΔΔCt^ method (Livak and Schmittgen, 2001).

### Plasmid Construction and Plant Transformation

The *SmMYB113* coding sequence was inserted into the pRI 101 vector containing the *35S-CaMV* promoter. The fusion vector was transferred into *Agrobacterium* strain LBA4404 and introduced into cultivar No. 108. The cut-cotyledons were pre-cultured on MS medium in the dark for 2 days. Then the cotyledons were infected with *Agrobacterium* (OD600 about 0.6) for 15–20 min. The infected-cotyledons were placed on the differentiation MS medium without Kan and co-cultured in the dark for 2–3 days. The differentiation MS medium consisted of 200 mg L^–1^ carbenicillin (Cb), 3.0 mg L^–1^ 6-benzylaminopurine (6-BA), 0.1 mg L^–1^ thidiazuron (TDZ), 7 g L^–1^ agar, 30 g L^–1^ sucrose, and 100 mg L^–1^ kanamycin. After the shoots were differentiated, the explants were placed on MS medium with 200 mg L^–1^ carbenicillin (Cb) for roots differentiation.

## Results and Discussion

### The Anthocyanin Contents in the Peels of Six Different Eggplant Cultivars

Eggplant is a widespread vegetable bearing different colored peels (Koley et al., 2019). Anthocyanins are the main phenolic compounds in eggplant peels. To explore the relationship between anthocyanin composition and color in eggplant, six eggplant cultivars representing all eggplant cultivar colors were examined (Duan et al., 2021). As shown in Supplementary Figure 1A, the six peel colors covered lavender in No. 44, reddish-purple in No. 64, black-purple in No. 76, white in No. 108, orange in No. 109, and green in No. 133. Peels samples were collected during fruit setting (fruiting), rapid growth (growth), and commodity maturity (maturity), and their relative anthocyanin contents were measured (Supplementary Figure 1B). Anthocyanin content increased along with fruit development. The anthocyanin contents were highest in cultivar No. 76, followed by No. 64, and No. 44. The anthocyanin contents of cultivars No. 133, No. 109, and No. 108 were the lowest, and did not differ significantly. These results indicated that higher anthocyanin contents corresponded to a deeper purple peel color, consistent with results in the distinct purple leaves of the novel tea cultivar ‘Ziyan’ (Lai et al., 2016).

### The Delphinidin/Flavonoid Ratio Could Better Explain the Purple Peel Color of Eggplants

**FIGURE 1:**
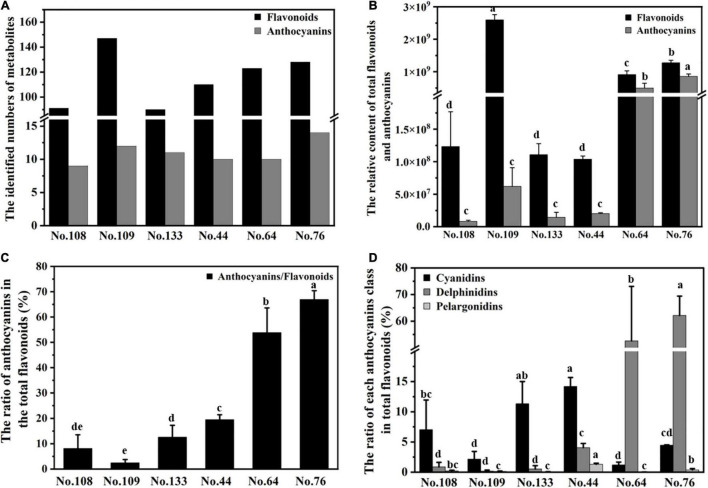
Overviews of the flavonoid-targeted metabolism data from the peels of six eggplant cultivars. **(A)** The identified number of total flavonoids and anthocyanins; **(B)** the relative contents of total flavonoids and anthocyanins; **(C)** the ratio of anthocyanins in the total flavonoids; **(D)** the ratio of cyanidins, delphinidins, and pelargonidins in the total flavonoids, respectively. Values are means ± SD (*n* = 3), same as following. Means denoted by the same letter did not differ significantly at *P* < 0.05 according to Duncan’s multiple range test.

Anthocyanin biosynthesis is a branch of flavonoid biosynthesis, and colored flavonoids (flavones, flavanols, and isoflavonoids) and their glycosides contribute to the diversity of colors in leaves, fruits, and flowers (Ono et al., 2010; Zhang et al., 2014; Shi J. et al., 2021). In eggplant, anthocyanins accumulated with fruit development and peaked at fruit maturity (Supplementary Figure 1). Therefore, flavonoid-targeted metabolism analysis was conducted on methanolic extracts of mature eggplant peels using UPLC-MS/MS. A total of 167 flavonoids were identified and divided into eight categories, including proanthocyanidins, anthocyanins, catechin derivatives, flavanone, flavone, flavone C-glycosides, flavonol, and flavonolignan (Supplementary Table 1 and Figure 1A). Among the 167 flavonoids, 16 anthocyanins, including 10 in No. 44, 10 in No. 64, 14 in No. 76, 9 in No. 108, 12 in No. 109, and 11 in No. 133, were identified. These results suggested that the eggplant cultivars without purple peels can also synthesize anthocyanins. Further analysis showed that the relative total flavonoid content ranked from highest to lowest as No. 109 > No. 76 > No. 64 > No. 44, No. 133, No. 108. The relative anthocyanin content was highest in No. 76, followed by No. 64, and No. 44, No. 133, No. 109, No. 108 (Figure 1B). Notably, the relative flavonoid content of No. 109 was greater than the other eggplant cultivars, but it contained almost no anthocyanin. Approximately 50% of the flavonoids in No. 64 and 70% of the flavonoids in No. 76 were anthocyanins (Figure 1C), resulting in purple peels. According to the “anthocyanins biosynthesis pathway (00942)” in the Kyoto Encyclopedia of Genes and Genomes (KEGG), the identified anthocyanins consisted of “cyanidins” (including cyanidins and peonidin derivatives), “delphinidins” (including delphinidins, malvidins, and petunidin derivatives), and “pelargonidins.” The delphinidin/flavonoid ratios of No. 76, No. 64, and No. 44 (purple peels), were higher than those of No. 133, No. 109, and No. 108 (no purple peels), particularly No. 76 and No. 64 (Figure 1D). Differently, the cyanidin/flavonoid ratios of No. 133, No. 109, and No. 108 were higher than No. 64 but lower than No. 44. The cyanidin/flavonoid ratio of No. 76 was higher than No. 109 but lower than No. 133 and No. 108. Together, a correlation was found between the delphinidin/flavonoid ratio and purple peel color among the six eggplant cultivars, meaning that the delphinidin/flavonoid ratio could better explain the purple coloration of eggplant peels.

Considering that No. 109 had the highest relative content of total flavonoids, but the lowest anthocyanin/flavonoid ratio, it was speculated that orange peel color resulted from flavones, flavanols, and isoflavonoids (Ono et al., 2010; Shi J. et al., 2021). As shown in Supplementary Figure 2A, the relative contents of flavones, flavanols, and isoflavonoids were highest in No. 109, followed by No. 76 and No. 64, No. 44 and No. 133, and No. 108. In addition, the ratio of flavones, flavanols, and isoflavonoids in the total flavonoids [(flavones and flavanols and isoflavonoids) / flavonoids)] was highest in No. 109 (Supplementary Figure 2B).

### *SmF3′5′H* Is Responsible for Purple Peel of Eggplant at the Transcriptional Level

**FIGURE 2:**
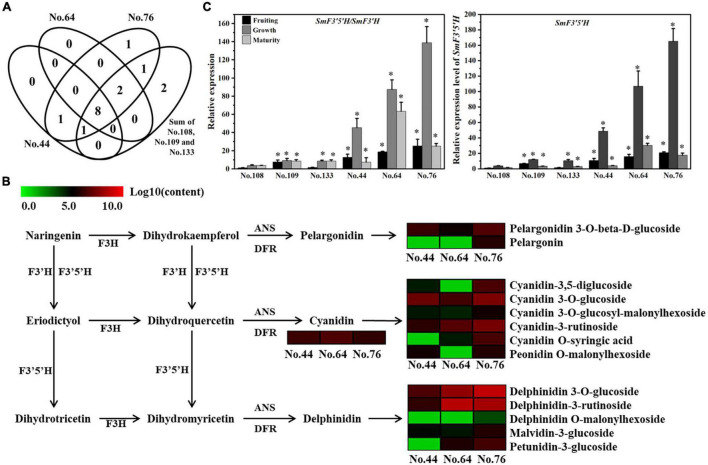
Veen analysis and heat map analysis of the identified anthocyanins, and the expression analysis of *SmF3′H* and *SmF3′5′H* in the peels of six eggplant cultivars. **(A)** Four-way Venn diagram analysis of the identified anthocyanins in the purple-peels colored eggplant cultivars with the sum of identified anthocyanins in the other three eggplant cultivars without purple peels color; **(B)** heat map of anthocyanins biosynthesis pathway. This pathway is constructed based on the KEGG pathway and literary references. Each colored row represents the log10 (content) of a metabolite; **(C)** the transcript ratio of *SmF3′H* and *SmF3′5′H*, and the relative expression level of *SmF3′5′H* in the peels of six eggplant cultivars at fruiting, growth and maturity stages. * represents significance at *P* < 0.05 comparing with those in No. 108.

To find the direct mechanism underlying the different purple peel colors of No. 76, No. 64, and No. 44, the 16 anthocyanin structures were further analyzed according to the anthocyanin biosynthesis pathway in the website of KEGG. Eight anthocyanins were found in the six eggplant cultivars, while “pelargonin” was found only in No. 76 and “cyanidin 3,5-O-diglucoside” was found both No. 76 and No. 44 (Figure 2A). Therefore, it was speculated that the eight anthocyanin structures cause purple peel colors. In the peels of No. 76 and No. 64, delphinidin 3-O-glucoside (Mirtillin) and “Tulipanin” contents were highest (Figure 2B and Supplementary Table 1). In the peels of No. 44, “Cyanidin 3-O-glucoside (Kuromanin)” content was highest, followed by “Mirtillin,” “Cyanidin,” and “Pelargonidin 3-O-beta-D-glucoside.” Based on the anthocyanin biosynthesis pathway in the KEGG website, we concluded that the “delphinidin” biosynthetic branch was significantly more active than the “cyanidin” and “pelargonidin” biosynthetic branches in No. 76 and No. 64, while the cyanidin biosynthetic branch activities were the strongest in No. 44. Flavonoid 3′-hydroxylases (F3′Hs) and flavonoid 3′,5′-hydroxylases (F3′5′Hs) competitively control the biosynthesis of cyanidin and delphinidin (Bogs et al., 2005; Castellarin et al., 2005). Castellarin et al. (2007) found that F3′H expression did not show a clear pattern associated with anthocyanin accumulation, but variation in the F3′5′H/F3′H expression ratio was consistent with anthocyanin biosynthesis in grape varieties. Therefore, the expression levels of *SmF3′H* and *SmF3′5′H* in the peels of eggplant fruits were analyzed using qRT-PCR. The expression ratio between *SmF3′5′H* and *SmF3′H* was also calculated. The *SmF3′5′H*/*SmF3′H* expression ratio was associated with the anthocyanin profiles variation among the six eggplant cultivars (Figure 2C). Notably, no significant difference in the relative expression level of *SmF3′H* was found among the six eggplant cultivars. However, *SmF3′5′H* expression level during the growth stage was highest in No. 76, followed by No. 64, No. 44, No. 133 and No. 109, and No. 108, consistent with the purple peel color pattern. Altogether, we speculated that the variation in purple peels of eggplant were mainly determined by the “Mirtillin” and “Tulipanin” contents, and that *SmF3′5′H* expression was a critical factor.

### Anthocyanins Accumulate in Eggplants After *SmMYB113* Overexpression

**FIGURE 3:**
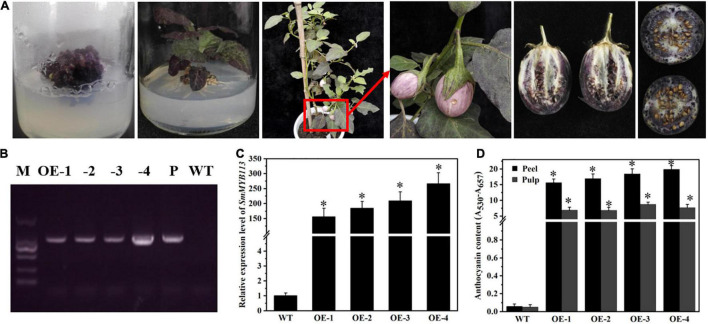
*SmMYB113* controls anthocyanin biosynthesis in eggplants. **(A)** The process of eggplant transformation and phenotypes of *SmMYB113*-OE eggplant line; PCR verification **(B)** and expression analysis **(C)** of *SmMYB113* in the four *SmMYB113*-OE eggplant lines. M represents 2,000 bp marker; P represents positive control using the *pRII-SmMYB113* vector; WT represents negative control using the DNA of No. 108; **(D)** the anthocyanin content in the peels and pulps of WT and the four *SmMYB113*-OE eggplant lines. Asterisk represents significance at *P* < 0.05 comparing with WT.

*SmMYB113* is an important regulator promoting anthocyanin biosynthesis in eggplant (Li et al., 2017; Shi S. et al., 2021). The eggplant *SmMYB113* gene was grouped with homologous R2R3MYBs from other plant species, including *AtPAP1* from *Arabidopsis* (Borevitz et al., 2000), *AmRosea1* and *AmDelila* from *Antirrhinum majus* (Sharma et al., 2020), *AN2* from petunia (Quattrocchio et al., 1999), *DcMYB113* from carrots (Xu et al., 2019), and *OsMYB3* from black rice cultivar (Zheng et al., 2021). Deletion of R2R3MYBs homologous results in tissue color loss (without anthocyanin enrichment or decrease), while overexpression results in more vivid coloration (anthocyanin enrichment or increase). Here, qRT-PCR analysis showed that *SmMYB113*expression levels in cultivars No. 108, No. 109, and No. 133 were significantly lower than those in No. 44, No. 64, and No. 76, corresponding to their anthocyanin contents (Supplementary Figure 3A). The *SmMYB113*full-length coding sequence was constructed into the overexpression pRI vector with the CaMV 35S promoter, obtaining *35S*:*SmMYB113* transgenic eggplant lines (Figure 3A and Supplementary Figure 3C). PCR and qRT-PCR analysis were used to confirm *SmMYB113* integration into the transgenic eggplant lines (Figures 3B,C). Compared with the WT, all the aboveground plant parts were purple. Anthocyanin contents measurement confirmed that the *SmMYB113-OE* plants produced more anthocyanins (Figure 3D). These results suggested that the *SmMYB113*function on regulating anthocyanin biosynthesis in eggplant is similar to that of R2R3MYB in other plant species.

### The Flavonoid Profiles in the Peels and Pulps of *SmMYB113-OE* Eggplants

**FIGURE 4:**
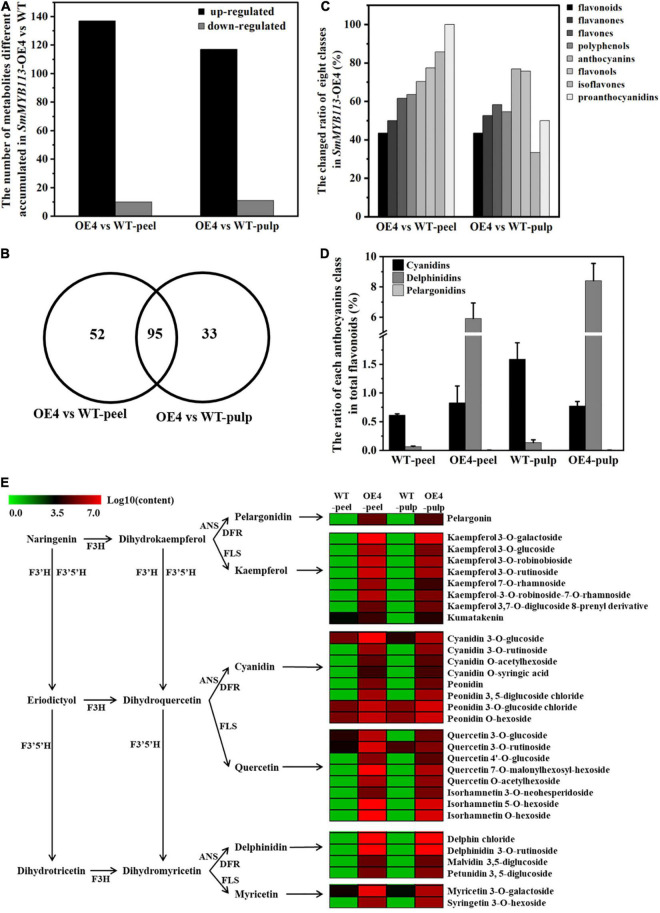
Overviews of the flavonoid-targeted metabolism data from the peels and pulps of *SmMYB113-*OE4, and heat map of flavonoid biosynthesis pathway. **(A)** The number of flavonoids different accumulated in the peels and pulps of *SmMYB113*-OE4 compared with WT at a level of |log_2_^(fold change)^| ≥ 1 and VIP ≥ 1; **(B)** Venn diagrams of different accumulated flavonoids by overexpression *SmMYB113* in the peels and pulps of eggplants; **(C)** the changed ratio of eight flavonoids classes by overexpression *SmMYB113*; **(D)** the ratio of cyanidins, delphinidins, and pelargonidins in the total flavonoids, respectively; **(E)** heat map of flavonoids biosynthesis pathway. This pathway is constructed based on the KEGG pathway and literary references. Each colored row represents the log10 (content) of a metabolite.

To reveal the flavonoid profiles regulated by *SmMYB113* in eggplant fruits, UPLC-MS/MS analysis was performed on the peels and pulps of WT and *SmMYB113-OE*4 plants. A total of 228 flavonoids were identified from peels and pulps, which could be classified into eight classes, including 23 flavonoids, 27 anthocyanins, 2 proanthocyanidins, 11 polyphenols, 91 flavones, 40 flavonols, 14 isoflavones, and 20 flavanones (Supplementary Table 2). Based on |log_2_^(fold change)^| ≥ 1 and VIP ≥ 1, 147 (in the peels) and 128 (in the pulps) flavonoids differed significantly between *SmMYB11*3-OE4 and WT plants. Most flavonoids contents were primarily increased *via SmMYB113* overexpression, except for 10 in peels and 11 in pulps which were downregulated (Figure 4A). More than 70% of anthocyanins and flavonols changed significantly *via SmMYB113* overexpression in both peels and pulps (Figure 4C), including 95 flavonoids changed in both peels and pulps, 52 in peels only, and 33 in pulps only (Figure 4B). Compared with WT, the delphinidin/flavonoid ratio in peels and pulps increased significantly in *SmMYB113*-OE4 (Figure 4D). Delphinidin 3,5-diglucoside (Delphin chloride) and “Tulipanin” were the most significantly increased by *SmMYB11*3, and their contents were highest in both peels and pulps (Supplementary Table 2). In addition, *SmF3′H* and *SmF3′5′H* relative expression levels in peels and pulps of *SmMYB113*-OE4 and WT were analyzed using qRT-PCR (Supplementary Figure 4). The *SmF3′5′H*/*SmF3′H* expression ratio was significantly increased by overexpressing *SmMYB113*, associated with the increased delphinidin/flavonoid ratio.

According to the KEGG pathway, anthocyanins and flavonols are two neighboring branches of flavonoid biosynthesis, catalyzing dihydroflavonols *via* DFR and FLS, respectively (Figure 4E). Strikingly, most anthocyanins and flavonols biosynthesis depends on *SmMYB113*. Similarly, following the knockout of *OsMYB3* in black rice, 12 anthocyanins were completely undetected (Zheng et al., 2021). These results implied that R2R3MYBs play a critical role in anthocyanin biosynthesis. In addition, the anthocyanins in *SmMYB113*-OE4 were primarily glycosylated at the 5-position of the B-ring, leading to the formation of highly stable and soluble anthocyanidin 3,5-diglucoside, such as delphinidin 3,5-diglucoside, malvidin 3,5-diglucoside, peonidin 3,5-diglucoside, and petunidin 3,5-diglucoside. Similarly, Sharma et al. (2020) reported that cyanidin 3-xylosyl(sinapoylglucosyl)galactoside was the main anthocyanin present in *AmRosea1*- and *AmDelila*-overexpressed taproots, while cyanidin 3-xylosyl(feruloylglucosyl)galactoside was the main anthocyanin present in the black carrot cultivar ‘Deep Purple’. The study of R2R3MYBs promoting anthocyanin biosynthesis has mainly focused on regulating structural gene expression (Karppinen et al., 2021), however, little is known about the regulation of modified genes.

The genes encoding glycosylation-modifying enzymes, including *Sm3GT* and *Sm5GT*, were analyzed according to the KEGG pathway annotation. Firstly, *Sm3GT* and *Sm5GT* relative expression levels in the peels and pulps of *SmMYB113*-OE4 and WT lines were analyzed by RT-qPCR (Supplementary Figures 5A,B). The results showed that *Sm3GT* and *Sm5GT* expression levels in the peels and pulps of the *SmMYB113*-OE4 line were significantly increased compared to WT. Subsequently, yeast one-hybrid assays (YIH) assay were performed between SmMYB113 and *Sm3GT* and *Sm5GT* promoters. However, no transcriptional regulation was found (Supplementary Figure 5C).

### Comprehensive Comparison of the Two Metabolomic Data in Eggplant

**FIGURE 5:**
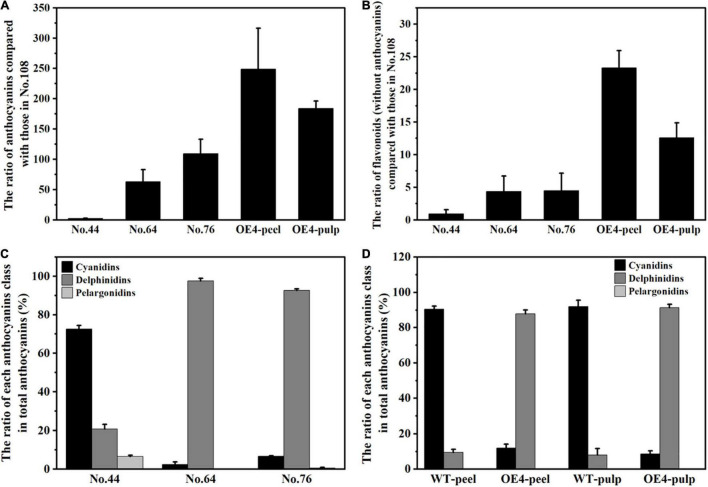
Comprehensive comparison of the two metabolomic data in eggplant. **(A)** The ratio of anthocyanins in the peels or pulps of three purple peels colored cultivars and *SmMYB113*-OE4 compared with those in No. 108; **(B)** the ratio of flavonoids (without anthocyanins) in the peels or pulps of three purple peels cultivars and *SmMYB113*-OE4 compared with those in No. 108; the ratio of cyanidins, delphinidins, and pelargonidins in the total anthocyanins in the peels three purple peels colored cultivars **(C)** and in the peels and pulps of *SmMYB113*-OE4 **(D)**.

Although the two metabolomic analyses were not performed simultaneously, the peels of No. 108 were included in both, and the metabolomics data could be connected by calculating the relative content of each sample to reference No. 108. The relative anthocyanin contents of purple peel cultivars No. 44, No. 64, and No. 76 were approximately 2-, 63-, and 109-fold greater than the anthocyanin content of No. 108. However, the relative anthocyanin contents in the peels and pulps of *SmMYB113*-OE4 were approximately 249- and 183-fold greater than the anthocyanin content of No. 108 (Figure 5A). This implied that molecular genetic breeding technology could make supernatural phenomena, one major advantage of molecular genetic breeding. However, the contents of the other flavonoids (flavonoids without anthocyanins) in the peels of purple cultivars No. 44, No. 64, and No. 76 were approximately 1-, 4.4-, and 4.5-fold greater than in No. 108. The other flavonoid contents in the peels and pulps of *SmMYB113*-OE4 were approximately 23- and 13-fold greater than in No. 108 (Figure 5B). Therefore, the anthocyanin biosynthesis regulation could affect the biosynthesis of other flavonoids. In addition, the amounts of the other flavonoids in No. 64 and No. 76 were similar (Figure 5B), suggesting that the different anthocyanin contents could mainly explain the color differences between No. 64 and No. 76. Furthermore, the anthocyanin structures in No. 64 were found in No. 76, and most anthocyanins’ content in No. 64 was less than in No. 76 (except “Tulipanin” and cyanidin) (Supplementary Table 1). Since cyanidin is an upstream product in anthocyanin biosynthesis, and the “Tulipanin” content was the highest in the peels of No. 64, we speculated that “Tulipanin” directly causes the reddish-purple peel coloration in No. 64.

Further analysis showed that the delphinidins were the main anthocyanins in the peels of No. 64, No. 76, and *SmMYB113*-OE4, and the pulps of *SmMYB113*-OE4 (Figures 5C,D), indicating that *SmMYB113* was indeed a critical transcript factor regulating anthocyanin biosynthesis in eggplant. However, the delphinidins structures differed between No. 64/No. 76 and *SmMYB113*-OE4 (Figures 2B, 4E). Compared with No. 64 and No. 76, *SmMYB113* overexpression induced anthocyanins glycosylation at the 5-position of the B-ring. Similar results were reported by Sharma et al. (2020), who reported that overexpression of *AmRosea1* and *AmDelila* (R2R3-MYB and bHLH TFs from *A. majus*) in taproots resulted in cyanidin 3-xylosyl(sinapoylglucosyl)galactoside accumulation and not cyanidin 3-xylosyl(feruloylglucosyl)galactoside accumulation. Although R2R3-MYB played a critical role in anthocyanin accumulation in plant tissues, other anthocyanin regulators also establish anthocyanin biosynthesis equilibrium. R2R3-MYB overexpression in plants cannot change the main anthocyanin class. However, it can disrupt their equilibrium by activating or enhancing the expression of downstream modifier genes, resulting in anthocyanin structure change.

In addition, previous studies have reported that delphinidin-3-(p-coumaroylrutinoside)-5-glucoside(nasunin) (Noda et al., 2000; Azuma et al., 2008), “Tulipanin” (Sadilova et al., 2006; Azuma et al., 2008; Todaro et al., 2009; Ferarsa et al., 2018; Yong et al., 2019; Condurache et al., 2021), “Mirtillin” (Azuma et al., 2008), delphinidin-3-[4-(cis-p-coumaroyl)-rhamnosyl-glucopyranoside]-5-glucopyranoside, and delphinidin-3-[4-(trans-p-coumaroyl)-rhamnosyl-glucopyranoside]-5-glucopyranoside (Li et al., 2017) were the major anthocyanins in eggplants with purple peels. Although the anthocyanin structures identified by these authors differed, they are all delphinidins.

## Conclusion

Anthocyanins are important chemical components leading to the purple coloration of eggplant peels. This study used targeted metabolic profiling of flavonoids to investigate the flavonoid and anthocyanin structures of six eggplant cultivars with different peel colors. This method detected 167 flavonoid metabolites, including 16 anthocyanins, with various modifications. The 16 anthocyanins could be classified in cyanidins, delphinidins, and pelargonins. According to our data, the purple color of eggplant peels was positively correlated with the delphinidin/flavonoid ratio, and “Mirtillin” and “Tulipanin” were the major anthocyanins in the purple eggplant peels. In addition, *SmF3′5′H* expression level in eggplant peels was sufficient to explain the purple color, so the *SmF3′5′H*/*SmF3′H* expression ratio was unnecessary. Simultaneously, targeted metabolic profiling of flavonoids was performed on the fruit peels and pulps of *SmMYB113* overexpressing eggplant lines. *SmMYB113* overexpression significantly increased the anthocyanins and flavonols contents in peels and pulps. Compared with WT, the delphinidin/flavonoid ratio was significantly increased, while no difference or small decreases in the cyanidin/flavonoid and pelargonin/flavonoid ratios were found. The delphinidins significantly accumulated as a result of *SmMYB113* overexpression in this study differed slightly from the data reported in other studies. Altogether, our data provide a glimpse into the flavonoid metabolites in eggplant peels with different colors and *SmMYB113-*overexpressed eggplant peels.

## Data Availability Statement

The datasets presented in this study can be found in online repositories. The names of the repository/repositories and accession number(s) can be found in the article/Supplementary Material.

## Author Contributions

GY: investigation and data curation. LL: investigation. MW: project administration. JL: project administration, data curation, software, and writing – original draft, review and editing. FY: project administration, and writing – review and editing. All authors contributed to the article and approved the submitted version.

## Conflict of Interest

The authors declare that the research was conducted in the absence of any commercial or financial relationships that could be construed as a potential conflict of interest.

## Publisher’s Note

All claims expressed in this article are solely those of the authors and do not necessarily represent those of their affiliated organizations, or those of the publisher, the editors and the reviewers. Any product that may be evaluated in this article, or claim that may be made by its manufacturer, is not guaranteed or endorsed by the publisher.

## Footnotes

1
http://www.shimadzu.com.cn/

2
http://www.appliedbiosystems.com.cn/

## Supplementary Material

Supplementary Figure 1 Fruit colors and the relative anthocyanins content of the six eggplant cultivars (Duan et al., 2021). **(A)** Fruit colors of the six eggplant cultivars at the commodity maturity stage; **(B)** the relative anthocyanins content in the eggplant peel at fruit setting (fruiting), rapid growth period (growth), and commodity maturity stage (maturity). Values are means ± SD (*n* = 3), same as following.

Supplementary Figure 2 Analysis of the colored flavonoids content in the peels of six eggplant cultivars. **(A)** The relative contents of the flavones, flavanols and isoflavonoids; **(B)** the ratio of the flavones, flavanols, and isoflavonoids in the total flavonoids.

Supplementary Figure 3 Expression analysis of *SmMYB113* and the phenotypes of WT and the four *SmMYB113*-OE eggplant lines. **(A)** The relative expression level of *SmMYB113* in the peels of six eggplant cultivars at fruiting, growth and maturity stages; the phenotypes of WT **(B)** and the four *SmMYB113*-OE eggplant lines **(C)**.

Supplementary Figure 4 Expression analysis of *SmF3′H* and *SmF3′5′H* in the peels and pulps of *SmMYB113*-OE4 line and WT.

Supplementary Figure 5 Expression analysis of *Sm3GT* and *Sm5GT* and transcriptional regulation analysis between SmMYB113 and *Sm3GT*, *Sm5GT*. The relative expression levels of Sm3GT **(A)** and *Sm5GT* **(B)** in the peels and pulps of *SmMYB113*-OE4 line and WT; **(C)** yeast one-hybrid assays indicating SmMYB113 could not bind the promoters of *Sm3GT* and *Sm5GT*. The combinations of p53-pABAi and 53-pGADT7 were used as positive control.
