# Supplementary figures and images for: SmMYB113 Is a Key Transcription Factor Responsible for Compositional Variation of Anthocyanin and Color Diversity Among Eggplant Peels
Source: Front Plant Sci. 2022 Mar 7;13:843996. doi: 10.3389/fpls.2022.843996 (PMC8959879; doi:10.3389/fpls.2022.843996)

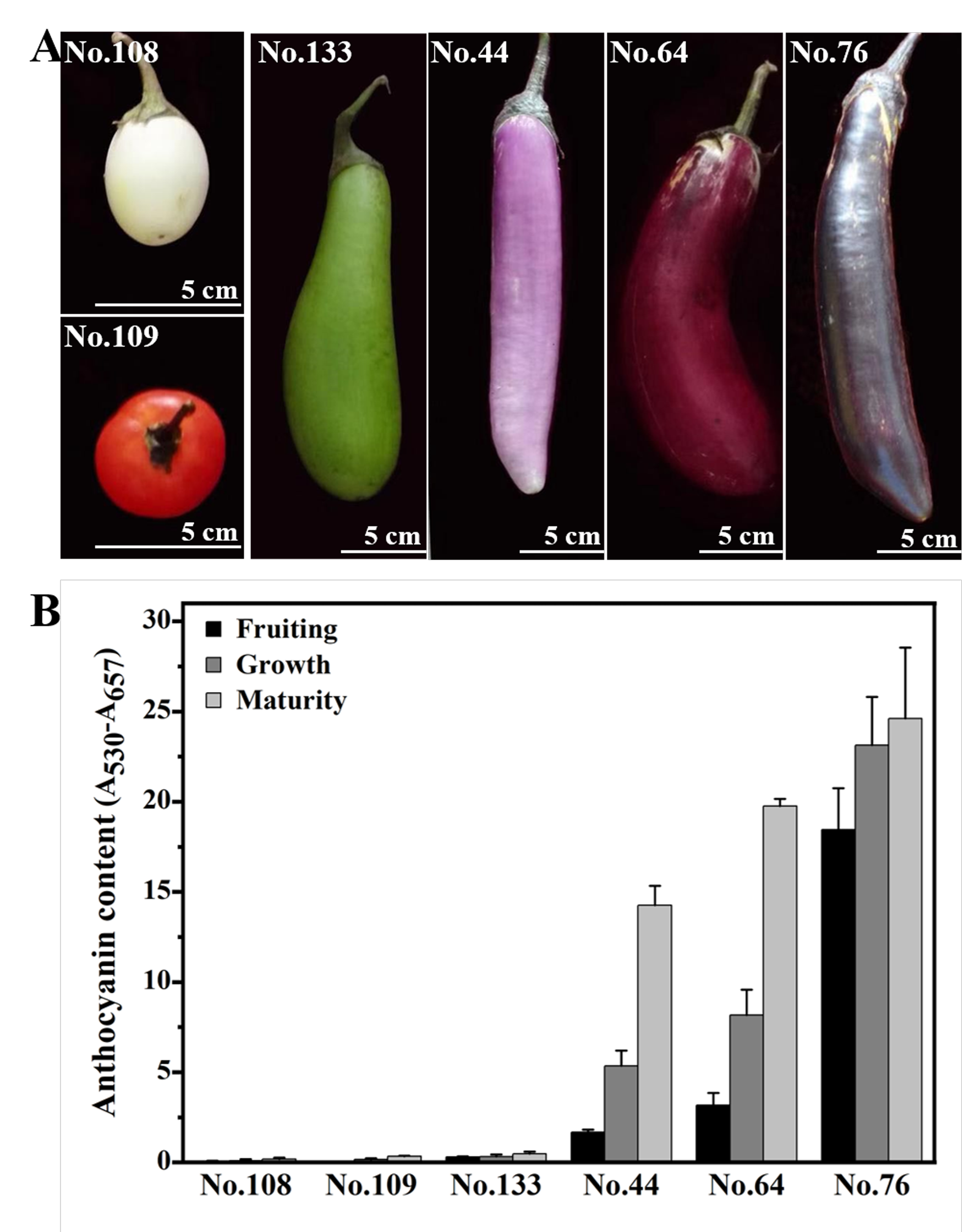

Supplement: Supplementary Figure 1 — Fruit colors and the relative anthocyanins content of the six eggplant cultivars (Duan et al., 2021). (A) Fruit colors of the six eggplant cultivars at the commodity maturity stage; (B) the relative anthocyanins content in the eggplant peel at fruit setting (fruiting), rapid growth period (growth), and commodity maturity stage (maturity). Values are means ± SD (n = 3), same as following. [file Image_1.TIF]

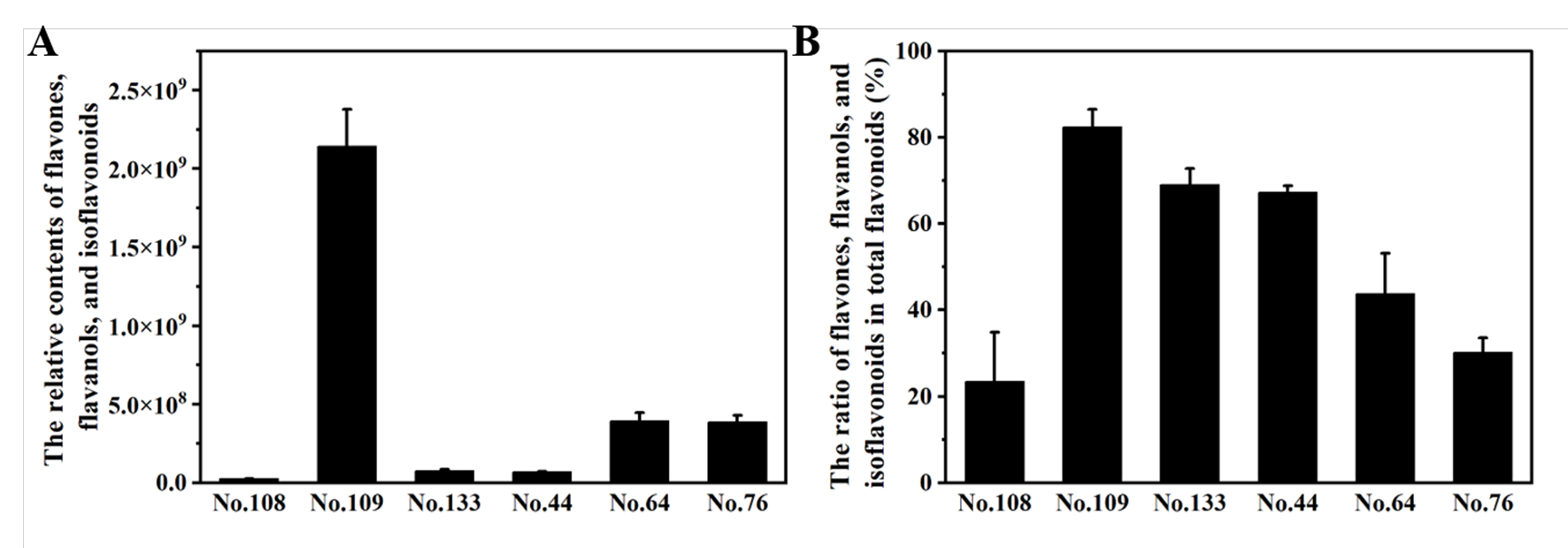

Supplement: Supplementary Figure 2 — Analysis of the colored flavonoids content in the peels of six eggplant cultivars. (A) The relative contents of the flavones, flavanols and isoflavonoids; (B) the ratio of the flavones, flavanols, and isoflavonoids in the total flavonoids. [file Image_2.TIF]

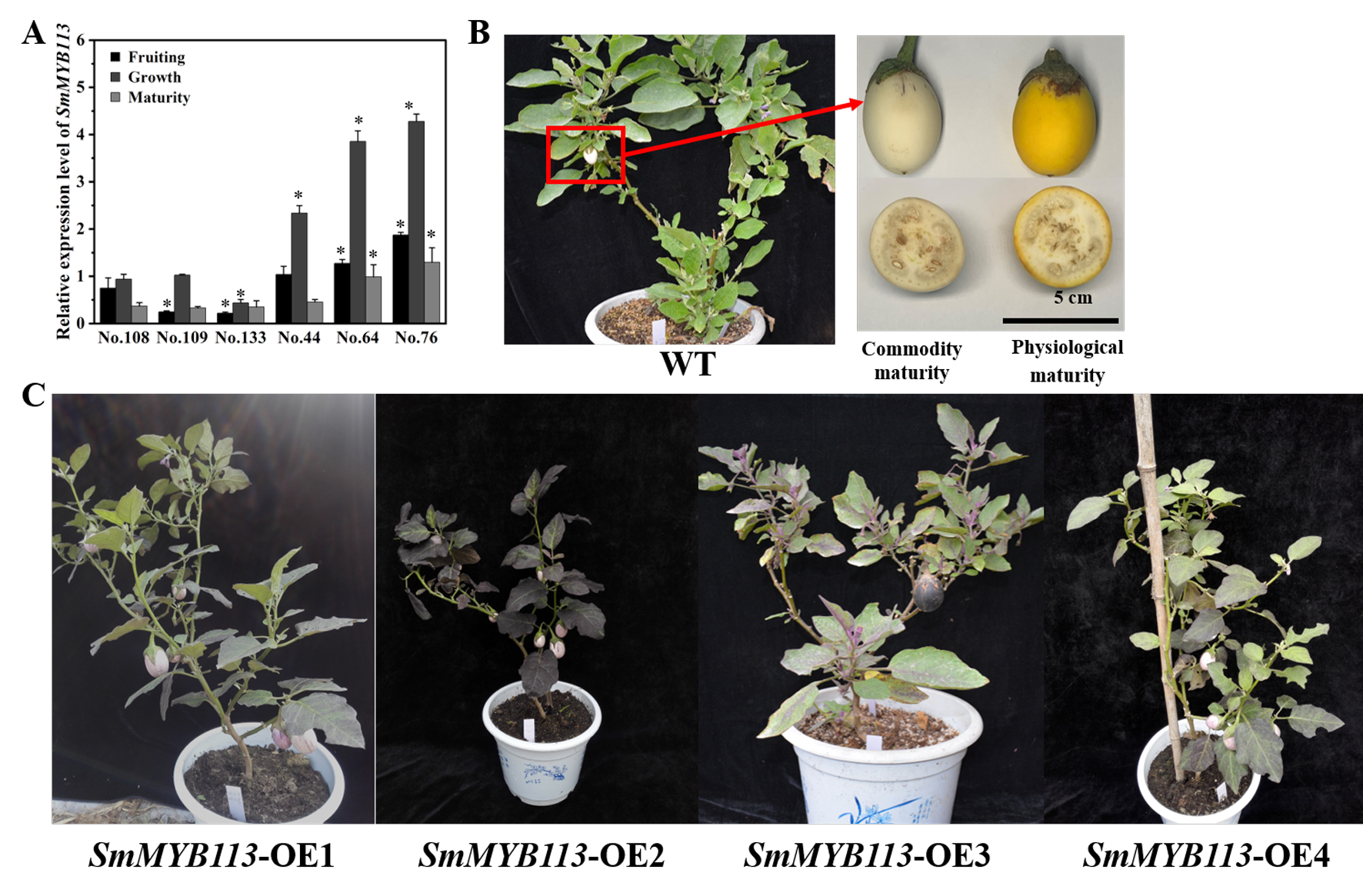

Supplement: Supplementary Figure 3 — Expression analysis of SmMYB113 and the phenotypes of WT and the four SmMYB113-OE eggplant lines. (A) The relative expression level of SmMYB113 in the peels of six eggplant cultivars at fruiting, growth and maturity stages; the phenotypes of WT (B) and the four SmMYB113-OE eggplant lines (C). [file Image_3.TIF]

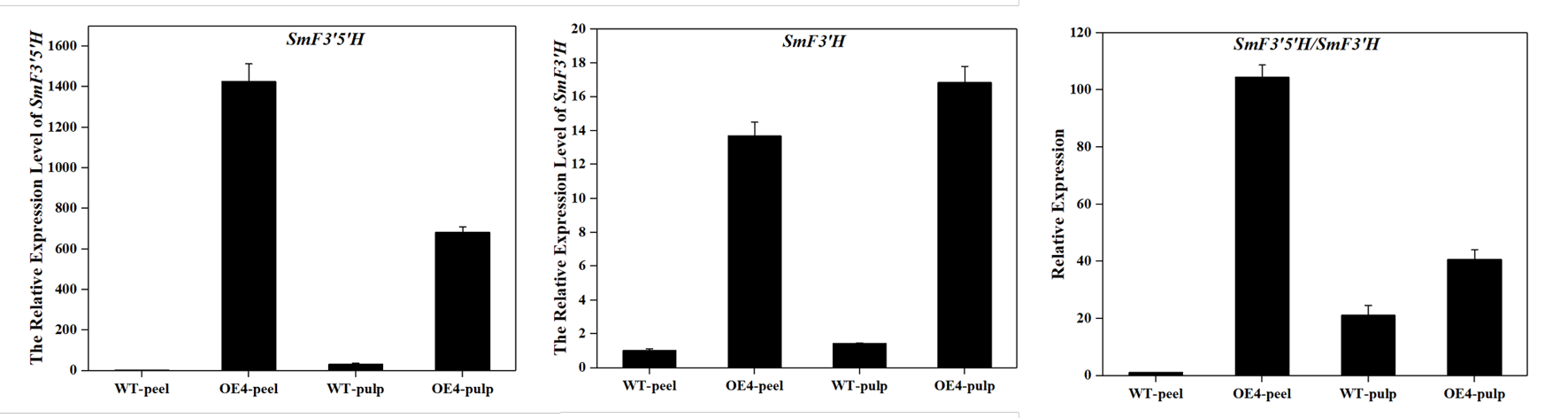

Supplement: Supplementary Figure 4 — Expression analysis of SmF3′H and SmF3′5′H in the peels and pulps of SmMYB113-OE4 line and WT. [file Image_4.TIF]

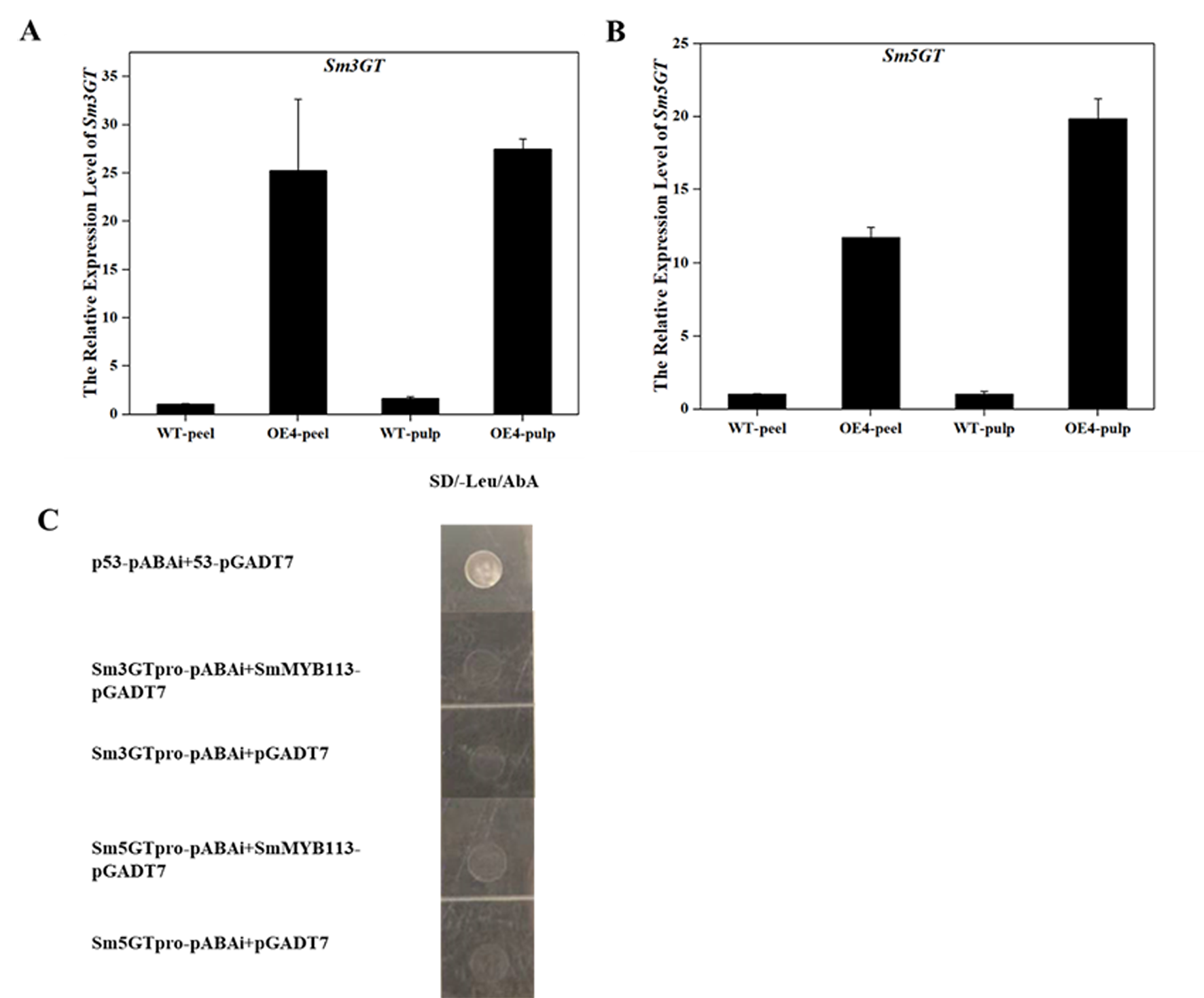

Supplement: Supplementary Figure 5 — Expression analysis of Sm3GT and Sm5GT and transcriptional regulation analysis between SmMYB113 and Sm3GT, Sm5GT. The relative expression levels of Sm3GT (A) and Sm5GT (B) in the peels and pulps of SmMYB113-OE4 line and WT; (C) yeast one-hybrid assays indicating SmMYB113 could not bind the promoters of Sm3GT and Sm5GT. The combinations of p53-pABAi and 53-pGADT7 were used as positive control. [file Image_5.TIF]
